# Follow-up attendance and documented reasons for non-attendance in child and adolescent mental health care: a retrospective analysis

**DOI:** 10.3389/fpsyt.2026.1927279

**Published:** 2026-09-04

**Authors:** Serkan Suren, Deniz Yavuz Baskiran

**Affiliations:** 1 Serkan Süren Child and Adolescent Psychiatry Clinic, Samsun, Türkiye; 2 Liver Transplantation Institute, Faculty of Medicine, Inonu University, Malatya, Türkiye

**Keywords:** adverse drug reactions, child and adolescent psychiatry, polypharmacy, retrospective study, treatment adherence

## Abstract

**Background:**

Failure to continue scheduled follow-up is a common challenge in child and adolescent mental health services and may reduce treatment effectiveness.

**Objective:**

The present retrospective study aimed to examine demographic, clinical, and treatment-related characteristics associated with attendance at the last scheduled follow-up appointment among children and adolescents receiving outpatient psychiatric care and to identify characteristics associated with the documented reasons for non-attendance among patients who missed their last scheduled follow-up appointment.

**Methods:**

A retrospective study was conducted using medical records of 841 patients aged 1–18 years who attended a tertiary child and adolescent psychiatry outpatient clinic between 2015 and 2026. Demographic, clinical, and treatment-related characteristics were summarized descriptively. Associations between categorical variables were examined using Pearson’s chi-square test, and effect sizes were reported using Cramér’s V.

**Results:**

Of the 841 patients, 142 had no scheduled follow-up appointment and were excluded from the attendance analysis. Among the remaining 699 patients, 56.8% attended their last scheduled follow-up appointment. No significant associations were observed between attendance and the demographic, clinical, or treatment-related characteristics examined. Among the 302 non-attendees, unspecified reasons (36.8%) and scheduling conflicts (17.5%) were most frequent. Documented reasons for non-attendance were significantly associated with medication prescription status (χ² = 9.749, p = 0.008, Cramér’s V = 0.181) and drug class (χ² = 10.202, p = 0.037, Cramér’s V = 0.130).

**Conclusions:**

Last scheduled follow-up attendance was not significantly associated with the demographic, clinical, or treatment-related characteristics examined. Among non-attendees, documented reasons for non-attendance were associated with medication prescription status and drug class. Systematic documentation of non-attendance reasons may help identify barriers to continuity of care and inform targeted follow-up strategies.

## 1 Introduction

Treatment adherence is a fundamental component of child and adolescent mental health services because the long-term course of psychiatric disorders such as attention-deficit/hyperactivity disorder (ADHD), anxiety disorders, obsessive-compulsive disorder (OCD), and autism spectrum disorder (ASD) largely depends on regular follow-up and continuity of care (1–3). Early discontinuation of follow-up has been associated with symptom recurrence, functional deterioration, increased healthcare utilization, and greater family burden (4, 5). Nevertheless, non-attendance rates for follow-up appointments in child and adolescent mental health services frequently exceed 50%, making the identification of factors contributing to treatment discontinuation a major priority for both clinical practice and research (6, 7). Numerous factors influencing continuity of follow-up have been investigated in the literature and can generally be classified into three categories: (i) patient- and family-related characteristics (e.g., age, sex, socioeconomic status, and previous psychiatric treatment history), (ii) clinical characteristics (e.g., diagnosis, illness severity, psychiatric comorbidity, and treatment complexity), and (iii) treatment-related factors (e.g., pharmacological treatment, adverse drug reactions, perceived treatment benefit, and the therapeutic alliance) (6–8). Existing reviews indicate that treatment discontinuation in child and adolescent mental health care is influenced by multiple interacting factors and that findings vary according to study design and the operational definition of dropout. A meta-analytic review of child and adolescent outpatient mental health care reported that dropout rates differed substantially between efficacy and effectiveness studies and that treatment-, therapist-, and participation-related barriers generally showed stronger associations with dropout than pretreatment child or family characteristics (4). More recently, a systematic review among youth with severe and enduring mental health problems identified treatment type, engagement, transparency and communication, goodness of fit between the young person and treatment, and practitioner-related perspectives as particularly relevant factors associated with treatment failure and dropout (7). Together, these findings suggest that discontinuation cannot be explained solely by demographic or diagnostic characteristics and should also be examined in relation to treatment processes, family engagement, and service-level factors. However, there is no consensus regarding the relative contribution of these factors to follow-up continuity. While some studies have identified demographic and sociocultural characteristics as the primary determinants (9), others have suggested that illness severity, treatment intensity, and adverse drug reactions play a more influential role (7). Adverse drug reactions (ADRs) are of particular interest because they represent potentially modifiable factors. However, evidence regarding the association between ADRs and treatment adherence in children and adolescents remains inconsistent. Some studies have reported that adverse effects such as weight gain or behavioral activation substantially reduce treatment adherence (10, 11), whereas others have found that this association is no longer significant after controlling for the type of medication prescribed (12). The present retrospective study aimed to examine demographic, clinical, and treatment-related characteristics associated with attendance at the last scheduled follow-up appointment among children and adolescents receiving outpatient psychiatric care, to describe the documented reasons for non-attendance, and to identify characteristics associated with these reasons among patients who missed their last scheduled follow-up appointment.

### 1.1 Research questions

RQ1. Which demographic, clinical, and treatment-related characteristics are associated with attendance at the last scheduled follow-up appointment among children and adolescents receiving outpatient psychiatric care?

RQ2. What are the documented reasons for missing the last scheduled follow-up appointment among patients who did not attend?

RQ3. Among patients who missed their last scheduled follow-up appointment, are the documented reasons for non-attendance associated with sociodemographic and treatment-related characteristics?

## 2 Methods

### 2.1 Study design and setting

This retrospective observational study was conducted at the outpatient child and adolescent psychiatry clinic of a tertiary referral center. The clinic provides comprehensive psychiatric assessment, pharmacological treatment, and brief psychotherapeutic interventions for children and adolescents referred by families, schools, and primary healthcare services. A retrospective observational design was considered appropriate because the study aimed to evaluate follow-up attendance and treatment discontinuation using electronic medical records collected during routine clinical practice (13). This study was reported in accordance with the Strengthening the Reporting of Observational Studies in Epidemiology (STROBE) Statement (14).

### 2.2 Study population

The study included all patients aged 1–18 years who attended at least one psychiatric outpatient evaluation between 2015 and 2026. The study period was defined from 2015 onwards because the electronic medical record system was implemented and maintained consistently from that year, allowing standardized retrieval of clinical information throughout the study period. No exclusion criteria were applied regarding psychiatric diagnosis, comorbidity, or treatment status. Prior to analysis, electronic medical records were reviewed for completeness and consistency. Patients with incomplete medical records that did not contain sufficient information for the variables included in the study were excluded. Consequently, 841 patients met the study eligibility criteria and were included in the final analyses.

### 2.3 Data collection

Demographic, clinical, and treatment-related information was extracted retrospectively from the electronic medical records using a structured data collection form. Variables included sex, age group, place of residence, presenting complaint, primary psychiatric diagnosis, psychiatric comorbidity, medication prescription, medication group, adverse drug reactions, referral source, administration of standardized psychological tests, number of outpatient visits, first and last visit dates, follow-up duration, attendance at the last scheduled appointment, and the documented reason for missing the last appointment. Psychiatric diagnoses were established according to the Diagnostic and Statistical Manual of Mental Disorders, Fifth Edition (DSM-5). For descriptive analyses, medication regimens were classified as stimulant monotherapy, antidepressant monotherapy, antipsychotic monotherapy, polypharmacy (≥2 medication classes), or no pharmacological treatment.

### 2.4 Outcome variable

The primary outcome was attendance at the last scheduled outpatient follow-up appointment. Attendance was recorded as a binary variable (attended/not attended) according to the medical records. For patients without a scheduled follow-up appointment, attendance at the last scheduled follow-up was considered not applicable, and these patients were excluded from the attendance analysis. Follow-up appointments were scheduled by the treating psychiatrist according to the patient’s clinical condition and routine clinical practice. Therefore, the interval between appointments was not fixed but reflected routine clinical care. For patients who missed their last scheduled appointment, the documented reason for non-attendance was extracted from the medical records. For descriptive analyses, the documented reasons were grouped into three categories: treatment-related reasons, social reasons, and unspecified reasons. Treatment-related reasons included perceived clinical improvement, treatment resistance or inadequate treatment response, refusal of pharmacological treatment, lack of parental motivation, and medication side effects, whereas social reasons included scheduling conflicts, transportation problems, patient or family illness, financial difficulties, and social events (e.g., weddings or funerals). Reasons that were not recorded in the medical records were classified as unspecified.

### 2.5 Statistical analysis

Continuous variables were summarized as mean ± standard deviation (SD), whereas categorical variables were presented as frequencies and percentages. Associations between categorical variables and last appointment attendance were examined using Pearson’s chi-square test, and effect sizes were reported as Cramér’s V. To further characterize missed follow-up appointments, selected sociodemographic and clinical variables (sex, comorbidity, medication prescription status, adverse effects, and drug class) were compared according to the documented reasons for missing the last scheduled follow-up appointment using Pearson’s chi-square test. Because medication classes included sparse categories, they were collapsed into three clinically meaningful groups (monotherapy, polypharmacy/other, and non-pharmacological treatment) for this analysis. Analyses were conducted using available cases for each variable; therefore, analytic denominators varied slightly because of missing data. Statistical significance was defined as p < 0.05. All statistical analyses were performed using IBM SPSS Statistics version 26.0 (IBM Corp., Armonk, NY, USA).

### 2.6 Ethical considerations

The study was conducted in accordance with the principles of the Declaration of Helsinki. Ethical approval was obtained from the İnönü University Non-Interventional Clinical Research Ethics Committee (Decision No. 2026/10411, Session Date: 16 June 2026). As this was a retrospective study based on routinely collected clinical records, the requirement for informed consent was waived by the ethics committee.

## 3 Results

A total of 841 patients were included in the study. The mean follow-up duration was 612 days [median, 312 days; interquartile range (IQR), 52–868 days], and the mean number of outpatient visits was 7.1 (median, 5; IQR, 2–10). Of the 841 patients, 142 had no scheduled follow-up appointment and were therefore excluded from the analysis of last scheduled follow-up attendance. Among the remaining 699 patients, 397 (56.8%) attended their last scheduled follow-up appointment, whereas 302 (43.2%) did not attend.

### 3.1 Demographic and clinical characteristics

**Table 1** summarizes the demographic, clinical, and treatment characteristics of the sample. The study sample was predominantly male (68.0%) and school-aged (72.4% aged 7–18 years). The most common primary diagnosis groups were neurodevelopmental disorders (33.4%), ADHD (30.3%), and anxiety/depression/OCD disorders (29.5%). Comorbidity was present in 62.4% of patients. Regarding treatment, 36.5% received monotherapy, 50.7% received polypharmacy/other treatment, and 12.8% received non-pharmacological treatment.

**Table 1:** Demographic, clinical, and treatment characteristics of the study sample (N = 841).

Characteristic	*n (%)*	Characteristic	*n (%)*
**Sex**		**Drug class (Multiple responses were allowed)**	
Male	572 (68.0)		
Female	269 (32.0)	Monotherapy	307 (36.5)
**Age group**		Polypharmacy/other	426 (50.7)
1–3 years	53 (6.3)	Non-pharmacological treatment	108 (12.8)
4–6 years	179 (21.3)	**Follow-up appointment status**	
7–11 years	314 (37.3)	Yes	397 (47.2)
12–18 years	295 (35.1)	No	302 (35.9)
**Primary diagnosis**		No scheduled follow-up	142 (16.9)
ADHD	255 (30.3)		
Anxiety/Depression/OCD disorders	248 (29.5)	**Side effect reported**	
Neurodevelopmental disorders	281 (33.4)	Yes	216 (25.7)
Mood and psychotic disorders	7 (0.8)	No	500 (59.5)
Other psychiatric disorders	15 (1.8)	Not applicable	125 (14.9)
No diagnosis	35 (4.2)	**Follow-up duration (days)**	
		No follow-up scheduled	142 (16.9)
**Comorbidity**		2–5 weeks	120 (14.3)
Yes	525 (62.4)	6–8 weeks	99 (11.8)
No	316 (37.6)	3–5 months	92 (10.9)
**Psychological testing**		≥6 months	388 (46.1)
Yes	381 (45.3)	**Continuous variables**	**Mean ± SD**
No	460 (54.7)	Number of visits	7.14 ± 6.89

*n*, number of patients; %, percentage; SD, standard deviation; OCD, Obsessive-Compulsive Disorder; SLD, Specific Learning Disorder; ASD, Autism Spectrum Disorder. Percentages may not total 100% due to rounding. Bold text is used to distinguish variable/category headings.

### 3.2 Reasons for missing the last follow-up appointment

**Table 2** summarizes the documented reasons for missing the last scheduled follow-up appointment among the 302 patients who did not attend. The reason for non-attendance was not documented in 36.8% of cases. Among cases with a documented reason, scheduling conflicts were the most frequently reported (17.5%), followed by transportation problems and clinical improvement (7.6% each), social reasons (7.0%), and patient or family illness (6.6%). Medication side effects accounted for only 1.3% of missed appointments.

**Table 2:** Documented reasons for missing the last scheduled follow-up appointment among patients who missed their last appointment (N = 302).

Characteristic	*n*	*%*
Scheduling conflict	53	17.5
Transportation problems	23	7.6
Clinical improvement	23	7.6
Social reasons (e.g., wedding, funeral)	21	7.0
Patient or family illness	20	6.6
Treatment resistance / inadequate treatment response	12	4.0
Refusal of pharmacological treatment	11	3.6
Lack of parental motivation	12	4.0
Financial reasons	12	4.0
Medication side effects	4	1.3
Not specified	111	36.8

*n*, number of patients; %, percentage.

### 3.3 Bivariate analyses

Of the 841 patients included in the study, 142 had no scheduled follow-up appointment and were therefore excluded from the analysis of last scheduled follow-up attendance. **Table 3** presents the bivariate associations between study variables and last scheduled follow-up attendance among the 699 patients with a scheduled follow-up appointment. No significant associations were observed between last scheduled follow-up attendance and sex, age group, comorbidity, primary diagnosis group, medication prescription, adverse effects, drug class, or psychological testing (all p > 0.05).

**Table 3:** Bivariate associations between study variables and last scheduled follow-up attendance (N = 699).

Variable		Attended n (%)	Did not attend n (%)	χ²	p	Cramér's V
Sex	Male	263 (55.4)	212 (44.6)	1.230^a^	0.267	0.042
	Female	134 (59.8)	90 (40.2)			
Age group	1–3 years	20 (50.0)	20 (50.0)	3.237^a^	0.357	0.068
	4–6 years	90 (59.6)	61 (40.4)			
	7–11 years	157 (59.5)	107 (40.5)			
	12–18 years	130 (53.3)	114 (46.7)			
Comorbidity	Yes	261 (56.7)	199 (43.3)	0.005^a^	0.945	0.003
	No	131 (56.5)	101 (43.5)			
Primary diagnosis group	ADHD	127 (57.2)	95 (42.8)	1.688^a^	0.890	0.049
	Anxiety/Depression/ OCD disorders	111 (54.4)	93 (45.6)			
	Neurodevelopmental disorders	136 (57.9)	99 (42.1)			
	Mood and psychotic disorders	5 (71.4)	2 (28.6)			
	Other psychiatric disorders	7 (53.8)	6 (46.2)			
	No diagnosis	11 (64.7)	6 (35.3)			
Medication prescribed	Yes	356 (57.5)	263 (42.5)	0.722^a^	0.396	0.032
	No	36 (52.2)	33 (47.8)			
Adverse Effects	Yes	110 (57.9)	80 (42.1)	0.080^a^	0.777	0.011
	No	242 (56.7)	185 (43.3)			
Drug class	Monotherapy	144 (58.8)	101 (41.2)	1.259^a^	0.533	0.042
	Polypharmacy/other	216 (56.5)	166 (43.5)			
	Non-pharmacological treatment	37 (51.4)	35 (48.6)			
Psychological test performed	Yes	197 (59.7)	133 (40.3)	1.984^a^	0.159	0.053
	No	198 (54.4)	166 (45.6)			

Pearson's chi-square test was used to compare categorical variables. Effect sizes are presented as Cramér's V. Statistical significance was defined as p < 0.05. OCD, Obsessive-Compulsive Disorder; SLD, Specific Learning Disorder; ASD, Autism Spectrum Disorder; Analytic denominators varied because of missing data, sex and age group, n = 699; comorbidity, n = 692; primary diagnosis group, n = 698; medication prescription, n = 688; adverse effects, n = 617; drug class, n = 699; and psychological testing, n = 694.

### 3.4 Comparison of reasons for missing the last follow-up appointment

**Table 4** compares selected sociodemographic and treatment-related characteristics according to the documented reasons for missing the last scheduled follow-up appointment among the 302 non-attendees. Variables included in this analysis were selected based on their potential clinical relevance to treatment discontinuation and continuity of care. Among the variables examined, medication prescription status (χ² = 9.749, p = 0.008, Cramér’s V = 0.181) and drug class (χ² = 10.202, p = 0.037, Cramér’s V = 0.130) were significantly associated with the documented reason for non-attendance. Patients who were prescribed medication more frequently had treatment-related or practical/social reasons for non-attendance, whereas unspecified reasons were more common among patients who were not prescribed medication. Similarly, unspecified reasons were more frequent among patients receiving non-pharmacological treatment than among those receiving monotherapy or polypharmacy/other treatment. No statistically significant associations were observed for sex (p = 0.601), comorbidity (p = 0.702), or adverse effects (p = 0.862).

**Table 4:** Comparison of reasons for missing the last follow-up appointment according to sociodemographic and treatment characteristics (N = 302).

Variables		Treatment-related reasons	Practical/ social barriers	Unspecified reasons	χ²	p	Cramér's V
		n (%)	n (%)	n (%)			
Sex	Female	13 (14.4)	45 (50.0)	32 (35.6)	1.020^a^	0.601	0.058
	Male	22 (10.4)	111 (52.4)	79 (37.3)			
Comorbidity	Yes	25 (12.6)	103 (51.8)	71 (35.7)	0.707^a^	0.702	0.049
	No	10 (9.9)	51 (50.5)	40 (39.6)			
Medication prescribed	Yes	32 (12.2)	144 (54.8)	87 (33.1)	9.749^a^	**0.008**	0.181
	No	3 (9.1)	10 (30.3)	20 (60.6)			
Adverse Effects	Yes	10 (12.5)	45 (56.3)	25 (31.3)	0.297^a^	0.862	0.033
	No	23 (12.4)	98 (53.0)	64 (34.6)			
Drug class	Monotherapy	14 (13.9)	56 (55.4)	31 (30.7)	10.202^a^	**0.037**	0.130
	Polypharmacy/ other	18 (10.8)	89 (53.6)	59 (35.5)			
	Non-pharmacological treatment	3 (8.6)	11 (31.4)	21 (60.0)			

Pearson's chi-square test was used to compare categorical variables. Effect sizes are presented as Cramér's V. Statistical significance was defined as p < 0.05. Analytic denominators varied because of missing data: sex, n = 302; comorbidity, n = 300; medication prescription status, n = 296; adverse effects, n = 265; and drug class, n = 302. Bold p-values indicate statistically significant results (p < 0.05)

## 4 Discussion

In this retrospective study, 841 children and adolescents receiving care at a tertiary child and adolescent mental health outpatient clinic were evaluated. Among the 699 patients with a scheduled follow-up appointment, 56.8% attended their last scheduled follow-up appointment. None of the demographic, clinical, or treatment-related variables examined were significantly associated with last scheduled follow-up attendance. Furthermore, among the 302 patients who missed their last scheduled follow-up appointment, both medication prescription status and drug class were significantly associated with the documented reason for non-attendance. Treatment-related reasons and practical/social barriers were more frequently documented among patients who were prescribed medication, whereas unspecified reasons were more frequent among those who were not prescribed medication. Similarly, unspecified reasons were more frequent among patients receiving non-pharmacological treatment than among those receiving monotherapy or polypharmacy/other treatment. No significant associations were observed between the documented reason for non-attendance and sex, comorbidity, or adverse effects. The present findings suggest that reasons for missed follow-up appointments may reflect multiple patient-, treatment-, and service-related factors rather than a single underlying cause. These findings are generally consistent with previous studies demonstrating that treatment discontinuation in child and adolescent mental health services is a multidimensional phenomenon. Consistent with previous systematic reviews, demographic and diagnostic characteristics alone may be insufficient to explain continuity of care. Previous research has highlighted the potential importance of treatment-related and service-related factors (4, 7). Nevertheless, a recent systematic review identified the quality of the therapeutic alliance, patient and family engagement, transparent communication, and alignment between treatment expectations and therapeutic approaches as key determinants of treatment adherence (7). In addition, service-level and clinician-related factors have been shown to play an important role in continuity of care (17), suggesting that healthcare service characteristics not evaluated in the present study may also influence follow-up behavior. Similarly, the high rates of early treatment discontinuation reported in tertiary child and adolescent mental health services highlight the need for service models that promote continuity of care, particularly following the initial assessment (15). Taken together, these findings suggest that missed follow-up appointments should be considered not only in relation to patient-level clinical characteristics but also within the broader context of the treatment process, patient and family engagement, and the organization of mental health services.

The finding that both medication prescription status and drug class were associated with the documented reasons for missing the last scheduled follow-up appointment supports the view that missed appointments are influenced by multiple interacting factors rather than a single determinant. A recent retrospective cohort study also reported that initiation of pharmacological treatment at the first psychiatric visit was associated with greater treatment retention (16). This may partly explain why patients receiving medication have more frequent contact with healthcare services, providing greater opportunities to communicate treatment-related concerns during follow-up. Recent systematic reviews have shown that missed appointments result from the interaction of multiple overlapping factors, including transportation difficulties, competing life events, limited flexibility in access to services, and patients’ perceptions that follow-up is unnecessary or no longer beneficial (18, 19). In this context, the classification of transportation problems, social events such as weddings or funerals, and financial difficulties as social reasons in the present study is consistent with the existing literature. Furthermore, the more frequent documentation of treatment-related or social reasons among patients who were prescribed medication may reflect their greater contact with healthcare services, resulting in more detailed documentation of the reasons for missed appointments in clinical records. In contrast, the higher proportion of unspecified reasons among patients who were not prescribed medication may be related to more limited contact with healthcare providers or incomplete documentation of the reasons for non-attendance. Similarly, the higher proportion of unspecified reasons among patients receiving non-pharmacological treatment compared with those receiving monotherapy or polypharmacy/other treatment may reflect differences in treatment pathways and patterns of contact with mental health services. Previous research has also demonstrated that continuity of care in child and adolescent mental health services is influenced not only by patient characteristics but also by the organization of healthcare services and the structure of the care pathway (20). In contrast, adverse effects were not significantly associated with the documented reasons for non-attendance in the present study. This finding differs from previous studies reporting that adverse effects may contribute to poorer medication adherence and more negative treatment attitudes among children and adolescents receiving psychiatric treatment (21). The discrepancy may be attributable to differences in sample characteristics, the retrospective nature of the clinical records, or the classification of reasons for missed appointments. The proportion of undocumented reasons for non-attendance may reflect documentation bias, as reasons for non-attendance may have been recorded more consistently in certain clinical situations than in others.

This study has several limitations. Owing to its retrospective design, the observed associations should not be interpreted as causal relationships. In addition, because the study relied on routinely collected clinical records, some variables may have been incompletely or inconsistently documented. The presence of undocumented reasons for missed follow-up appointments may have introduced documentation bias and may have limited the robustness and generalizability of the observed associations, as the recorded reasons may not accurately reflect the full spectrum of factors underlying missed appointments. Furthermore, the study was conducted at a single tertiary referral center, which may limit the generalizability of the findings to primary care settings or other mental health service providers. The study covered an 11-year period during which changes in clinical practice, service organization, and external events such as the COVID-19 pandemic may have influenced follow-up attendance. In addition, patients without a scheduled follow-up appointment were excluded from the attendance analysis because this outcome was not applicable to these patients. These temporal factors could not be evaluated separately and therefore may have affected the observed patterns of continuity of care. Important factors that may influence continuity of care, including socioeconomic status, family functioning, parental attitudes toward treatment, the therapeutic alliance, and access to mental health services, were not available in the medical records and therefore could not be evaluated. Previous studies have shown that family characteristics, parental mental health, socioeconomic disadvantage, accessibility of mental health services, travel distance, and organizational factors may also influence continuity of care and should be considered in future research (22, 23). Future multicenter prospective studies incorporating psychosocial and healthcare service–related factors, together with more comprehensive and standardized documentation of missed appointments, are warranted to provide a more comprehensive understanding of the determinants of continuity of care in child and adolescent mental health services.

From a clinical perspective, the findings of this study have important implications for outpatient child and adolescent mental health services. Rather than treating all missed appointments as a homogeneous phenomenon, routinely documenting and evaluating the reasons for non-attendance may provide valuable information for clinical decision-making. In particular, systematically distinguishing treatment-related reasons, practical or social barriers, and undocumented reasons for non-attendance may facilitate the development of tailored follow-up strategies and improve continuity of care. Systematic assessment of the reasons for missed appointments may therefore contribute to improving continuity of care and to the more effective organization and delivery of child and adolescent mental health services.

## 5 Conclusion

In this retrospective study of 841 children and adolescents receiving outpatient psychiatric care, no significant associations were observed between last scheduled follow-up attendance and the demographic, clinical, or treatment-related characteristics examined. Among patients who missed their last scheduled follow-up appointment, medication prescription status and drug class were significantly associated with the documented reason for non-attendance. No significant associations were observed between the documented reason for non-attendance and sex, comorbidity, or adverse effects. These findings emphasize the importance of routinely documenting and evaluating the reasons for missed appointments in clinical practice. Identifying the underlying causes of appointment non-attendance may help improve continuity of care and inform targeted strategies to enhance follow-up in child and adolescent mental health services.

## Data Availability

The raw data supporting the conclusions of this article will be made available by the authors, without undue reservation.
